# TDP-43 pathology in a patient carrying G2019S *LRRK2* mutation and a novel p.Q124E *MAPT*^☆^

**DOI:** 10.1016/j.neurobiolaging.2013.04.011

**Published:** 2013-12

**Authors:** Helen Ling, Eleanna Kara, Rina Bandopadhyay, John Hardy, Janice Holton, Georgia Xiromerisiou, Andrew Lees, Henry Houlden, Tamas Revesz

**Affiliations:** Reta Lila Weston Institute of Neurological Studies and Queen Square Brain Bank for Neurological Disorders, Department of Molecular Neuroscience, Institute of Neurology, University College London, London, UK

**Keywords:** *LRRK2*, *MAPT*, Parkinson's disease, TDP-43, tau

## Abstract

Leucine-rich repeat kinase 2 (*LRRK2*) mutation is the most common cause of genetic-related parkinsonism and is usually associated with Lewy body pathology; however, tau, α-synuclein, and ubiquitin pathologies have also been reported. We report the case of a patient carrying the *LRRK2* G2019S mutation and a novel heterozygous variant c.370C>G, p.Q124E in exon 4 of the microtubule-associated protein tau (*MAPT*). The patient developed parkinsonism with good levodopa response in her 70s. Neuropathological analysis revealed nigral degeneration and Alzheimer-type tau pathology without Lewy bodies. Immunohistochemical staining using phospho-TDP-43 antibodies identified occasional TDP-43 pathology in the hippocampus, temporal neocortex, striatum, and substantia nigra. However, TDP-43 pathology was not identified in another 4 archival *LRRK2* G2019S cases with Lewy body pathology available in the Queen Square Brain Bank. Among other published cases of patients carrying *LRRK2* G2019S mutation, only 3 were reportedly evaluated for TDP-43 pathology, and the results were negative. The role of the *MAPT* variant in the clinical and pathological manifestation in *LRRK2* cases remains to be determined.

## 1 Introduction

Among the 5 identified pathogenic leucine-rich repeat kinase 2 (*LRRK2*) mutations, G2019S is the most common, and accounts for 1% of sporadic Parkinson's disease and 4% of hereditary parkinsonism worldwide (Healy et al., 2008). Clinical presentation of *LRRK2* mutation resembles idiopathic Parkinson's disease but may be associated with a more benign disease course (Healy et al., 2008). Most patients with *LRRK2* mutation exhibit neuropathological features consistent with typical Lewy body Parkinson's disease (Gilks et al., 2005; Khan et al., 2005; Zimprich et al., 2004); however, “pure” nigral degeneration, tau, α-synuclein, or ubiquitin pathologies resembling progressive supranuclear palsy (PSP), multiple system atrophy, and frontotemporal lobar degeneration with ubiquitin-positive inclusions (FTLD-U) have also been reported (Dachsel et al., 2007; Gaig et al., 2008; Giasson et al., 2006; Hasegawa et al., 2009; Rajput et al., 2006; Zimprich et al., 2004). Ubiquitinated TAR DNA-binding protein–43 (TDP-43) is a major disease protein in FTLD and amyotrophic lateral sclerosis (ALS) and can occasionally be observed in Lewy body disorders and tauopathies (Mackenzie et al., 2010; Neumann et al., 2006; Sreedharan et al., 2008). Recently, TDP-43–related pathology was reported in 3 patients carrying *LRRK2* mutations (p.R1441C, p.R793M and L1165P) (Covy et al., 2009; Wider et al., 2010). Here, we report a patient with a clinical diagnosis of Parkinson's disease, who in post-mortem was found to have nigral degeneration without Lewy body pathology, and was also shown to carry the *LRRK2* G2019S mutation and a novel heterozygous variant c.370C>G, p.Q124E in exon 4 of the microtubule-associated protein tau (*MAPT*).

## 2 Methods

### 2.1 Case selection and genetic analysis

Genomic DNA was extracted from frozen brain tissue of these cases, and Sanger sequencing was performed according to standard procedures as previously described (Kara et al., 2012). As part of an ongoing clinicopathological study to evaluate archival cases with post-encephalitic parkinsonism (PEP) and unclassifiable tauopathy at the Queen Square Brain Bank for Neurological Disorders (QSBB), we sequenced *LRRK2* (exons 24, 25, 27, 29, 35, 36, 41, and 48), *PARK2* and *MAPT* genes in 13 cases. *PARK2* was chosen because it can cause levodopa-responsive parkinsonism with absence of Lewy bodies (Doherty et al., 2013), whereas *LRRK2* mutations are more commonly associated with Lewy body pathology (Wider et al., 2010). *MAPT* gene was sequenced because of the presence of tau pathology in these cases. From this cohort, we recently published our findings of the rare *MAPT* p.A152T variant as a risk factor for the development of tauopathies (Kara et al., 2012). MRC-Holland (Amsterdam, the Netherlands) multiplex ligation-dependent probe amplification (MLPA) kits (P051, P052) were used for copy-number analysis of the following genes: *PARK2* 6q25.2, *SNCA* 4q21, *Pink1*, *Park7* 1p36, *UCHL1* 4p14, *GCH1* 14q22.1, and *LRRK2* 12q12. Mutations and variants identified in *MAPT, LRRK2,* and *PARK2* were named based on transcripts with accession numbers NM_005910.5/NP_005901.2, NM_198578.3/NP_940980.3, and NM_004562.2/NP_004553.2, respectively. *MAPT* haplotypes were determined by the genotype of the H1/H2-tagging SNP rs1052553 (Hayesmoore et al., 2009). The *TDP-43* gene was sequenced in the present case with TDP-43 pathology. In 4 other cases with proven *LRRK2* G2019S mutations and Lewy body pathology available in the QSBB (Gilks et al., 2005), sequencing of the *MAPT* gene was performed.

Subjects in this report had provided written consent to perform neuropathological and genetic studies. All protocols of brain donation had been approved by a London Research Ethics Committee, and tissue is stored for research at the QSBB with a license from the Human Tissue Authority. This research was approved by the Tissue Request Committee of the QSBB.

### 2.2 Neuropathology

Following the QSBB protocols, the brains were divided mid-sagittally post mortem. One-half of the brain was immediately frozen and stored at −80 °C, and the other half was immersed and fixed in 10% neutral formalin for 3 weeks. After slicing and sampling the brain, tissue blocks were processed using standard protocols. We performed hematoxylin and eosin, Luxol fast blue/cresyl violet, and Congo red staining on 7-μm-thick sections, and also used the modified Bielschowsky and Gallyas silver impregnation methods. Immunohistochemistry with antibodies to phospho-tau (AT8 clone recognizing Ser202/Thr205; BioScience Life Sciences; 1:600), 3-repeat (3R) and 4-repeat (4R) tau isoforms (Upstate/Millipore; 3R tau: RD3; 1:2000; 4R tau: RD4; 1:200) (de Silva et al., 2003), Aβ (Dako; 6F/3D; 1:100), ubiquitin, p62, TDP, p-TDP, α-synuclein (Vector Laboratories; KM51; 1:50) and phospho-α-synuclein (recognizing Ser 129; Abcam-ab59264; rabbit polyclonal) was carried out using a standard avidin–biotin method.

In 4 other cases with proven *LRRK2* G2019S mutations and Lewy body pathology available in the QSBB (Gilks et al., 2005), immunohistochemistry staining using antibody to phospho-TDP-43 (*p*-TDP) was carried out in the hippocampus, amygdala, and upper midbrain blocks.

To assess whether the neuronal loss in the substantia nigra pars compacta (SNpc) identified in the *LRRK2* case was related to the genetic mutation or to its Alzheimer's related pathology, we performed semi-quantitative assessment of nigral cell loss in 12 randomly selected cases with confirmed pathological diagnosis of Alzheimer's disease (AD).

## 3 Results

### 3.1 Genetic analysis

**Fig. 1:**
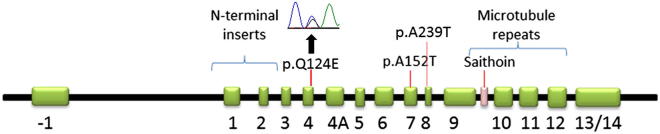
Diagram of the *MAPT* gene depicting the location of the 3 rare variants reported to date to be associated with tau pathology. The novel variant c.370C>G is indicated with a black arrow on the chromatogram above the p.Q124E variant.

Of the 13 cases with PEP and unclassifiable tauopathy we identified 1 case with both *LRRK2* G2019S mutation and a heterozygous variant in *MAPT* exon 4 (c.370C>G, p.Q124E) (Fig. 1). The *MAPT* p.Q124E variant is absent in all control subjects in the public databases (N = 6568, of which 3913 are caucasians), which include, Ensemble (http://useast.ensembl.org/index.html) and Exome Variant Server (Exome Variant Server, NHLBI GO Exome Sequencing Project (ESP), Seattle, WA (URL: http://evs.gs.washington.edu/EVS/) [(1,2013) accessed]) (see Supplementary Table 1); this variant is also absent in the 4 *LRRK2* cases with Lewy body pathology. The *MAPT* haplotype of this patient was H1/H2. *PARK2* and *TDP-43* sequencings in this case were negative. A dosage study using MLPA was negative for all analyzed genes including *PARK2* and *LRRK2*.

### 3.2 Case report

The British white woman who carried the *LRRK2* G2019S mutation and the heterozygous p.Q124E *MAPT* variant, developed right-hand tremor at age 73 years. She had mild bradykinesia and rigidity and was diagnosed with idiopathic Parkinson's disease. She was initially started on an anticholinergic medication; 5 years later, levodopa therapy was administered and titrated to 1000 mg/day, with a good response. She had no cognitive impairment. Her motor symptoms gradually deteriorated. She reported wearing-off symptoms but never had any dyskinesia. In the last year of life, she had balance difficulty, multiple falls and required a rollator to mobilize. She died at age 85. There was no family history of any movement or neurological disorders.

**Fig. 2:**
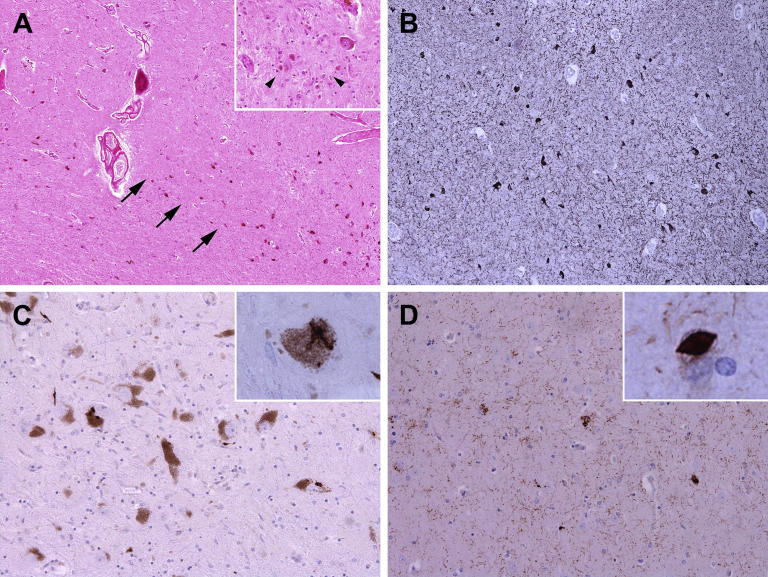
Key neuropathological findings in the present case. Severe loss of neuromelanin-containing neurons in the ventrolateral tier of the substantia nigra (arrows), as well as gliosis and free pigment (arrowheads) (A); Abundant tau-positive neurofibrillary tangles, neuropil threads, and pre-tangles in the entorhinal cortex (B); phospho-TDP-43 (*p*-TDP) immunoreactive neuronal cytoplasmic inclusions (NCIs), neurites, and a skein-like structure (inset) in the substantia nigra (C); and numerous *p*-TDP–positive fine thread-like processes, few coarser neurites, round, dot-like structures, and a “cat's-eye” neuronal intranuclear inclusion (NII) (inset) in the subiculum (D). A, hematoxylin and eosin staining; B, AT8; and C and D, phospho-TDP-43 immunostaining.

Neuropathological analysis confirmed an overall moderate degree of neuronal loss in the SNpc except in the ventrolateral tier, where severe neuronal loss was found (Fig. 2). There were no Lewy bodies on α-synuclein or phospho-α-synuclein immunohistochemistry. There were sparse tau-positive neuropil threads (NTs) in the frontal and parietal cortices; sparse NTs, neurofibrillary tangles (NFTs), and pre-tangles (PreTs) in the CA1 and CA4 hippocampal subregions; mild PreTs, moderate numbers of NPs, NFTs and NTs in the subiculum; severe NFTs, NTs, and PreTs in the entorhinal cortex and very sparse NTs in the striatum (Fig. 2). All tau inclusions were both 3R- and 4R-tau positive. The subthalamic nucleus was preserved, and no tau pathology was noted.

Aβ immunohistochemistry demonstrated parenchymal deposition in the cerebral cortex, hippocampus, and striatum corresponding to Thal phase score of 3 (Thal et al., 2002), whereas the distribution of tau pathology corresponded to Braak and Braak stage III (Braak et al., 2006). Using the Consortium to Establish a Registry for Alzheimer's disease (CERAD) criteria (Mirra et al., 1991), the NP score was “sparse” in the temporal cortex, “moderate” in the frontal cortex, and “sparse” in the parietal cortex. These results gave a “low” likelihood of AD, according to the National Institute on Ageing (NIA)/Reagan Institute of the Alzheimer Association Consensus Recommendations for the Postmortem Diagnosis of Alzheimer's disease, 1997 (NIA-Reagan criteria) (Hyman and Trojanowski, 1997), and “intermediate” level of AD pathologic change (A2, C2, B2) according to the 2012 guideline (Hyman et al., 2012).

TDP-43 and p-TDP-43 immunohistochemistry showed numerous fine thread-like processes and few coarser neurites in the CA1 hippocampal subregion and subiculum. In the amygdala and the temporal and frontal cortices, there were occasional NCIs and few threads. There were occasional NCIs, threads, and NIIs in the subiculum, striatum, and SN, and skein-like NCIs in the SN (Fig. 2).

There was no hippocampal sclerosis. No α-synuclein immunoreactive inclusions, argyrophilic grains, cerebral amyloid angiopathy (CAA), or vascular pathology was observed. No P62-positve “star-shaped” inclusion in the hippocampus or small “dot-like” structures in the cerebellar granule cells were observed.

### 3.3 Findings in other LRRK2 G2019S cases

In 4 other cases available in the QSBB with proven *LRRK2* G2019S mutation, no TDP-43–related pathology was observed in the hippocampus or amygdala. Sequencing of the *MAPT* gene did not reveal any abnormal finding.

### 3.4 Nigral degeneration in AD control cases

In the 12 randomly selected cases with confirmed pathological diagnosis of Alzheimer's disease (NIA/Reagan “high” likelihood of AD), nigral cell loss was, at most, mild, as evidenced by regional pigment incontinence in the SNpc.

## 4 Discussion

*LRRK2* G2019S mutation is commonly associated with Lewy body pathology. Of the 22 published post-mortem cases with this mutation, only 4 cases had an absence of Lewy bodies. Rajput et al reported a case with slow, progressive, non–levodopa-responsive parkinsonism and tau-positive NFTs resembling the neuropathology of PSP (Rajput et al., 2006), Giasson et al and Gaig et al each reported a case with classical tremor-dominant parkinsonism and pure nigral degeneration (Gaig et al., 2008; Giasson et al., 2006), and, Dachsel et al reported a case with dementia and tremor and pathology consisted of FTLD with ubiquitinated neuronal inclusions (FTLD-U) (Dachsel et al., 2007). On the other hand, pleomorphic pathologies including α-synuclein, tau, and ubiquitin seem to be more commonly associated with other pathogenic *LRRK2* mutations (Hasegawa and Kowa, 1997; Hasegawa et al., 2009; Santpere and Ferrer, 2009; Wszolek et al., 1997, 2004; Zimprich et al., 2004).

We report a case with *LRRK2* G2019S mutation clinically diagnosed as Parkinson's disease, with good levodopa response, in which neuropathological analysis revealed nigral degeneration with an absence of Lewy bodies, Alzheimer-type tau, and TDP-43 pathologies. Interestingly, this patient was also found to carry a novel p.Q124E *MAPT* variant. Prompted by the TDP-43 pathology, we screened this case for TDP-43 mutations, which were negative. We then surveyed for TDP-43 pathology in another 4 archival QSBB cases with *LRRK2* G2019S mutation and Lewy body pathology, and the findings were negative. These 4 *LRRK2* G2019S cases were also negative for pathogenic mutations or novel variants in *MAPT*. To date, only 3 cases with *LRRK2* mutation have been reported to have TDP-43–related pathology (p.R1441C, p.R793M, and L1165P) (Covy et al., 2009; Zimprich et al., 2004), none of which had the G2019S mutation. Only 3 other published cases carrying the *LRRK2* G2019S mutation were evaluated for TDP-43 pathology (Giasson et al., 2006) but no TDP-43–positive inclusions were observed (Covy et al., 2009).

The discovery of TDP-43 in 2006 as a major disease protein in FTLD and ALS led to the introduction of TDP-43 immunohistochemistry into the routine diagnostic protocols of brain banks in the last few years. It is therefore likely that other reported *LRRK2* cases with ubiquitin pathology may also have TDP-43 inclusions and will warrant comprehensive assessment (Dachsel et al., 2007; Wszolek et al., 1997, 2004; Zimprich et al., 2004). In addition to FTLD-TDP and ALS, TDP-43 inclusions can sometimes be detected in other neurodegenerative diseases including AD, Lewy body disorders, and primary tauopathies including corticobasal degeneration, PSP, parkinsonism-dementia complex of Guam, and dementia pugilistica (Arai et al., 2009; Hasegawa et al., 2007; King et al., 2010; Mackenzie et al., 2010; McKee et al., 2013; Uryu et al., 2008; Yokota et al., 2010). The cause and mechanism of TDP-43 in tauopathies are not known, but it has been postulated that tau aggregates may promote aggregation of TDP-43 through cross-seeding (Morales et al., 2009; Uryu et al., 2008). TDP-43 immunoreactivity may modify clinical features in AD and other types of dementia (Josephs et al., 2008; Lashley et al., 2011), and is also closely associated with hippocampal sclerosis (Amador-Ortiz et al., 2007; Josephs et al., 2008). It remains to be determined whether TDP-43 protein plays a role in influencing the clinical features in *LRRK2* cases. As in Lewy-body Parkinson's disease, nigral degeneration is a typical finding in *LRRK2* mutation and is considered to be the pathological substrate of clinical parkinsonism in these patients (Wider et al., 2010). Although previous studies have shown a correlation between nigral pathology and extrapyramidal symptoms in AD (Burns et al., 2005), our screening of 12 randomly selected AD cases did not reveal significant nigral cell loss, in contrast to the present *LRRK2* case. It is therefore unlikely that the modest Alzheimer-type tau pathology in this case would explain the extent of the nigral cell loss.

The pleomorphic pathologies in *LRRK2* mutation supports the notion that *LRRK2* acts upstream from the pathway of other proteins implicated in neuronal death; and it is likely that genetic and environmental factors then influence the type of proteinopathy that eventually develops in the individual, whether it is α-synuclein, tau, or ubiquitin pathology (Wider et al., 2010). The novel *MAPT* variant identified in our case is located in a region of the protein far from microtubule-binding domains and does not have an obvious role in the molecule's function. A similar variant was also recently reported in exon 7 of the *MAPT* gene (Coppola et al., 2012; Cruchaga et al., 2012; Jin et al., 2012; Kara et al., 2012), and a rare variant p.A239T in exon 8 (NM_005910.5) was found in a carrier of a C9orf72 repeat expansion (King et al., 2013) (Fig. 1). Interestingly, these 3 rare variants all localize in an uncharacterized region of the *MAPT* protein in cases with unexpected tau pathology, which supports the speculation that these variants may have a disease-modifying role and may predispose the individual to tau pathology (Coppola et al., 2012; Devine and Lewis, 2008; Gan-Or et al., 2012; Kara et al., 2012). However, the precise mechanism of this relation is far from clear, and our genetic findings in this case serve as an interesting observation rather than yielding a definitive conclusion. It is noteworthy that, in *LRRK2* cases with atypical clinical presentation and/or unusual pathologies, one should also consider the possibility of a coincidental neurodegenerative process with a non-penetrant *LRRK2* mutation (Goldwurm et al., 2011; Sierra et al., 2011; Xiromerisiou et al., 2012).

## Disclosure statement

None of the authors have potential or actual conflicts of interest, and all the authors have seen the manuscript before submission. The work was funded by the Reta Lila Weston Foundation and the PSP Brain Bank. The funding source had no role in study design, data collection and analysis, decision to publish, or preparation of the manuscript. There is no animal work in this study, and the human work on blood and pathology materials has been carried out in compliance with UK regulations.
